# A pilot evaluation of the role of a children’s wellbeing practitioner (CWP) in a child and adolescent mental health service (CAMHS)

**DOI:** 10.1177/13591045231157621

**Published:** 2023-04-14

**Authors:** Michael Turnbull, Hayley Kirk, Michealla Lincoln, Sarah Peacock, Lynne Howey

**Affiliations:** 1 Tees Esk and Wear Valley NHS Foundation Trust, West Park Hospital, Darlington, UK; 2 Faculty of Health and Life Science, 373117 Northumbria University, Newcastle Upon Tyne, UK; 3 Department of Clinical Psychology, University of Teesside, Middlesbrough, UK

**Keywords:** Children’s wellbeing practitioner, improving access to psychological therapies, low intensity cognitive behavioural therapy, anxiety, depression

## Abstract

**Background:**

In 2017, the Children and Young People’s Improving Access to Psychological Therapies (CYP-IAPT) project was extended to deliver low-intensity Cognitive Behavioural Therapy (CBT), delivered by Children’s Wellbeing Practitioners (CWPs), but to date evaluation is sparse.

**Aims:**

To evaluate low-intensity interventions delivered by trainee CWPs for the treatment of anxiety and depression in a child and adolescent mental health service (CAMHS).

**Method:**

The evaluation adopted a quantitative, within-subjects, cross-sectional design. The outcome measures of 98 service users aged 8–17 years were included in the evaluation. Service users were children and young people accessing CAMHS in the North East of England. Outcome measures included the Revised Children’s Anxiety and Depression Scale (RCADS-47) and Goal Based Outcomes (GBOs). Descriptive data relating to the types of interventions used and outcomes following CWP involvement were also explored.

**Results:**

Analysis of pre and post intervention data highlighted significant reduction in symptomatology across all RCADS subscales and composite total scales, and significant goal progress as measures by GBO’s. Effect sizes ranged from moderate to large (*d* = 0.75 – 0.90) across all subscales of the RCADS. Large effect sizes were found for depression, total anxiety and total RCADS scores (*d *= 0.86, *d* = 1.12, *d* = 1.14), and GBOs (*d* = −1.33).

**Conclusions:**

Findings support the potential value of low intensity CBT interventions delivered by CWPs in reducing anxiety and depression in this population. Recommendations for the development of the CWP role and CWP services are discussed.

## Introduction

In 2007, the UK government launched the Improving Access to Psychological Therapies (IAPT) initiative. This was a large-scale attempt to improve access to evidence-based psychological therapies for adults with depression and anxiety disorders (Department of Health, 2012). At its core, IAPT aimed to increase the delivery of psychological treatments in line with recommendations from the National Institute for Health and Clinical Excellence (NICE). In 2004, a systematic review of the evidence for the efficacy of CBT as a treatment for depression, anxiety, obsessive-compulsive disorder (OCD) and post-traumatic stress disorder (PTSD) was conducted by NICE. Following this review, guidance was published that advocated CBT as an effective treatment for these disorders (NICE, 2004a; 2004b). What remained, however, was a gap in the ability of existing mental health services to deliver services in line with such recommendations. IAPT was intended to fulfil this shortcoming by training and employing a new clinical workforce to deliver CBT interventions for the treatment of depression and anxiety.

NICE have advocated the value of low-intensity CBT interventions within a framework of stepped-care model for the treatment of mild to moderate depression and anxiety difficulties (NICE, 2004a; 2004b). Within this service delivery model treatments are offered in differing intensities by a range of professionals depending on the type and severity of mental health difficulty, and monitored by way of evaluating outcomes (Bower & Gilbody, 2005). The role of the adult Psychological Wellbeing Practitioner (PWP) is to deliver low-intensity CBT interventions such as guided self-help or psychoeducation (National Collaborating Centre for Mental Health, 2018). PWPs complete a postgraduate certificate lasting 1 year (The IAPT Manual) and act as ‘coaches’, rather than traditional therapists, who deliver self-help treatment protocols aligned with a low-contact high volume approach (Richards & Whyte, 2009). Interventions delivered by PWPs typically consist of 6–8 sessions. Therefore, those service users who require more high-intensity psychological therapies are stepped up to high intensity treatment accordingly (Care Services and Improvement Partnership Choice and Access Team, 2008). There is currently limited research exploring the efficacy of the PWP role in delivering low-intensity CBT interventions in line with the IAPT agenda (Van Straten et al., 2015). Other studies suggest that therapist effects accounted for 9% of the variance of service user outcomes, and indicated the influential role of symptom severity, treatment duration and attendance on outcomes (Firth et al., 2015); Green et al., 2014).

Nevertheless, initial evaluation data from IAPT demonstrator sites has shown promising findings including an improvement in recovery rates (55–56%) of anxiety and depression for those attending two or more appointments (Clark, et al., 2009). Similarly, the first phase of IAPT was found to meet the desired targets in relation to both the number of staff trained and the proportion of service users assessed (Clark, 2011). However, in spite of such findings, IAPT has not been without its critics. For example, some have argued that the IAPT initiative has failed to deliver in its aim to fulfil an agenda of parity of esteem for mental and physical health services (Department of Health, 2011). This is said to be in part due to the increasing need for psychological interventions and the barriers posed by waiting times and lack of patient choice reported (Mind, 2013). Furthermore, it has been suggested that there continues to be a mismatch between service need and what is delivered in practise as CBT treatment (Shafran et al., 2009). This provides an ongoing barrier to the implementation of IAPT.

In 2011, the Children and Young People’s IAPT programme (CYP-IAPT) aimed to improve services delivering mental health care to children, young people and their families (Department of Health, 2015). As a government funded agenda, CYP-IAPT aimed to improve access to evidence-based psychological therapies, enhance clinical outcomes and service user experience. In 2017, the CYP-IAPT initiative was extended to include the training of CWPs, to deliver evidence based (step 2) low-intensity CBT interventions to children and young people with mild to moderate depression and anxiety disorders including therapist guided self-help principles (GSH). This initiative aimed to free up existing high intensity specialist clinicians who were reportedly struggling to work through the volume of more complex cases within specialist CAMHS (Fonagy, 2019). The child model of delivery largely aimed to mimic the adult model in terms of target population, focus of intervention and the number of sessions being delivered. Whilst in its infancy, research data is currently sparse for attempts to evaluate the effectiveness of CYP-IAPT and more specifically the interventions delivered by CWPs (Fuggle & Hepburn, 2019). Ludlow et al., (2020) suggest that Low intensity CYP-IAPT interventions show promise however it is imperative that robust evaluation is implemented. Given the lack of published literature to date, it is currently difficult to make conclusions around the value of CWPs in implementing evidence-based interventions within the CYP-IAPT project.

In response to the conception of the CWP project, children and young people’s services in Tees Esk and Wear Valley NHS Foundation Trust began to transform services to expand access to evidence based low-intensity interventions for mild to moderate mental health problems. As part of this, a CWP pilot service was established with 5 trainee CWPs. Within the service model, referrals were screened via the Single Point of Access (SPA) into which all CWPs work 1 day a week. If referrals were deemed appropriate for CWP intervention, then they would be booked into CWP assessment and intervention slots. Interventions would typically last 6 – 8 sessions and be in relation to mild to moderate low mood and anxiety. CWPs received comprehensive Clinical Case Load Management and Clinical Skills Supervisions alternating weeks. This meant that CWPs had the opportunity to discuss cases with senior clinicians on a weekly basis and received continual skills-based development. Hence, this pilot service evaluation aims to start to help address the gap in the literature by evaluating the outcomes of trainee CWPs working within a real-world CAMHS setting.

## Research question and aims

The study has two primary aims which were as follows:

1. To assess if the interventions carried out by CWPs were meeting their intended aim of improving the self-reported severity of mood and anxiety disorders as measured by the Revised Children’s Anxiety and Depression Scale (RCADS).

The criterion for improvement was the statistically significant post-treatment decrease in the scores on the major depressive disorder (MDD), generalised anxiety disorder (GAD), obsessive compulsive disorder (OCD), panic disorder (PD), separation anxiety disorder (SAD), social phobia (SP) subscales, as well as total anxiety and total depression scores.

2. To determine if there were significant post-treatment improvements in the subjective assessment of progress made towards goals set by the child/young person.

The criterion for improvement was a significant increase in a quantitative Goal Based Outcomes assessment.

## Method

### Participants

The outcome measures of 98 service users, who had completed low-intensity CBT interventions, were included in the analysis. This data was taken from 216 referrals to the Single Point of Access that were assessed by CWPs, 103 of whom were deemed appropriate for CWP input and had completed a low-intensity CBT intervention by the time of analysis. All those who completed an evidence-based multi-session intervention completed pre- and post-intervention measures and were included in the analysis (*n* = 98). All 98 service users were under the care of CAMHS in the North East of England across four geographical locations. All had received low-intensity CBT interventions from five trainee CWPs as part of standard care (mean number of appointments offered = 7.57, mean number attended = 6.99). The age of participants ranged from 8 to 17 years (mean age = 14.02 standard deviation = 2.09) and the gender ratio between males and females was 24:74. Inclusion criteria for CWP intervention included service users with mild to moderate depression (and/or low-level self-harm) and/or anxiety.

For the remaining 118 cases from the 216 referrals descriptive data showed that service users were offered a mean number of 3.93 appointments and the main outcomes from referral were discharge to alternative provision or discharge with pure self-help (*n* = 68), ongoing support from trainee CWP’s (*n* = 27), step up to Tier 3 CAMHS (*n* = 17) and transfer to Tier 2 CAMHS (*n* = 6). Given the CWP role, pre- and post-outcome measures would not have been expected for these cases as they did not receive or had not finished a low-intensity CBT intervention.

### Measures

Two outcome measures collected as part of standard service practice were used to assess depression and anxiety symptoms and self-reported goal attainment pre- and post- CWP intervention. The outcome measures included The Revised Children’s Anxiety and Depression Scale (RCADS-47): Child report version and Goal Based Outcomes (GBO’s). GBOs were only recorded on electronic notes system for evaluation during the later part of the evaluation due to service implementation issues. Descriptive data relating to the type of interventions used, outcomes following CWP intervention as well as qualitative feedback from service users were also collected as part of the evaluation.

### The Revised Children’s Anxiety and Depression Scale (*RCADS-47*): Child report version

The RCADS is a 47 item self-report questionnaire that measures six subscales; major depressive disorder (MDD), generalised anxiety disorder (GAD), obsessive compulsive disorder (OCD), panic disorder (PD), separation anxiety disorder (SAD), social phobia (SP), as well as a total anxiety and total depression scores. Items are scored between 0-3 on a 4-point Likert scale which corresponds to responses of never, sometimes, often or always. The RCADS has been shown to demonstrate good internal reliability (Chronbach’s alpha = .93) in both clinical and non-clinical samples (Chorpita et al., 2000; Chorpita et al., 2005).

### Goal Based Outcomes

GBOs evaluate subjective progress made towards a goal set by the child or young person prior to an intervention during clinical work (Law & Jacob, 2015). Lower scores indicate distance from a set goal (0 = no goal progress) whereas higher scores represent progress towards the goal (10 = goal fully achieved).

### Procedure

Outcome data was collected from the electronic notes of 98 service users who had previously completed interventions delivered by trainee CWPs. Data was transferred from an excel database to an R data file for analysis.

### Data analysis

The data of 98 service users was analysed. Tests of normality using Shapiro Wilk revealed the data was not normally distributed (*p* < .001) therefore non-parametric statistics were adopted within the analysis. T-tests were used to analyse the difference between pre- and post-intervention data for the major depressive disorder (MDD), generalised anxiety disorder (GAD), obsessive compulsive disorder (OCD), panic disorder (PD), separation anxiety disorder (SAD), social phobia (SP), as well as a total anxiety and total depression scores on the RCADS. Aggregated progress scores of up to three GBOs were also compared using Wilcoxon signed rank tests. Apriori power calculations conducted for t-tests (one-tailed prediction) specified a minimum sample of 27 participants required for 0.8 power with a medium effect size (Cohen, 1992). Where noted, Cohen’s d values of less than 0.40 represent a ‘small’ effect, values between 0.40 and 0.80 reflect a ‘moderate’ effect, and values greater than 0.80 reflect a ‘large’ effect.

## Results

### Descriptive statistics

Table 1 shows the means and standard deviations of each RCADS-47 subscale score pre and post CWP intervention. Interventions delivered by CWPs included low intensity CBT for anxiety (*n* = 35), psychoeducation/guided self-help (*n* = 33), behavioural activation (*n* = 21), pure self-help with watchful waiting (*n* = 8) and parent-led CBT (*n* = 1). Outcomes following intervention comprised discharge from service (*n* = 80), step up to Tier 3 (*n* = 12), transfer to Tier 2 (*n* = 2) and ongoing support/second intervention from CWPs (*n* = 4).

**Table 1.** Descriptive Statistics and Comparison of pre- and post-treatment means for the RCADS and GBO’s.

RCADS-47 subscale	Pre-intervention mean (SD)	Post-intervention mean (SD)	V (df)	*p*	Cohen’s d
MDD	65.94 (18.39)	50.82 (16.63)	4224.5 (97)	<0.001	0.86
GAD	55.63 (11.59)	45.03 (11.93)	3700 (97)	<0.001	0.90
PD	69.63 (15.79)	57.09 (17.49)	3729 (97)	<0.001	0.75
OCD	54.33 (11.49)	44.60 (10.71)	3496 (97)	<0.001	0.88
SP	57.73 (12.49)	46.60 (12.37)	4066 (97)	<0.001	0.90
SAD	68.68 (14.40)	56.13 (14.12)	4117.5 (97)	<0.001	0.88
Total anxiety	64.54 (12.52)	49.24 (14.25)	4304 (97)	<0.001	1.14
Total RCADS	66.18 (13.85)	49.74 (15.50)	4561.5 (97)	<0.001	1.12
GBO *n* = 28	4.57 (3.66)	10.75 (5.49)	4 (27)	<0.001	−1.32

*Note.* SD = standard deviation, GBO = goal-based outcomes, *n* = 98 unless stated.

### Difference between pre and post intervention scores on the RCADS and goal progress (GBO’s)

Pre and post intervention data, shown in Table 1, highlighted significant improvements across RCADS and GBO’s. Effect sizes demonstrated moderate to large effects *(d* = 0.75 - 0.90) for pre to post changes for all subscales of the RCADS. Large effect sizes were also found for total anxiety and total RCADS scores (*d* = 1.12, *d* = 1.14). Findings demonstrate broad improvements across all mental health domains as measured using the RCADS following CWP intervention.

The pre and post intervention GBO data was analysed for a sample of 28 service users. A Wilcoxon signed rank test was used as the data was ordinal and not normally distributed (*p* < .05). A significant difference was found been pre and post goal-based outcomes (*p* < .001). Post intervention goals (M = 10.75, SD = 5.49) were significantly greater than pre-intervention goals (M = 4.57, SD = 3.66). Findings indicate that progress towards goals increased following CWP intervention.

## Discussion

The main findings of this service evaluation demonstrate that the interventions carried out by CWPs were meeting their intended aims in relation to improved depression and anxiety symptomatology and self-reported goal attainment. Firstly, the difference between pre and post intervention scores from the RCADS were found to be significant. Results showed a significant reduction in symptoms of depression and anxiety following low-intensity CBT interventions delivered by trainee CWPs across all six subscales; categorised by medium to large effect sizes. Such findings coincide with research demonstrating the effectiveness of CBT (Shafran et al., 2009), and supports the CYP-IAPT agenda and its aim to allow service users to return to a level of normal functioning (Department of Health, 2008a). This study goes some way to address the gap that remains in the current literature for evaluating the role of CWPs.

Outcomes from self-reported goal progress using GBOs suggested improvements post CWP intervention. This is in line with research that has shown the value of creating collaborative goals prior to treatment and how this may coincide with recovery (Law & Jacob, 2015). The low number of cases (*n =* 28) was due to systemic recording issues during the CWP service initiation and is acknowledged as a study limitation. Indeed, research has highlighted barriers to the emphasis on routine outcome monitoring as part of CYP-IAPT (Wolpert et al., 2012).

Operationally, findings showed that most service users were discharged from service following CWP intervention (*n* = 80) which supports the value of CYP-IAPT agenda in terms of meeting its intended aims to enhance clinical throughput in a cost-effective fashion (Department of Health, 2015). This is important given the current pressures noted in CAMHS to see service users quickly despite ever increasing referral rates and need for specialist mental health support. Furthermore, this highlights the importance of referral criteria for CWP intervention in the first instance to ensure that service users have access to the right level of treatment delivered by the most appropriate professional in line with the stepped model of care (Bower & Gilbody, 2005). Interventions carried out by CWPs included low-intensity CBT for anxiety, psychoeducation and guided self-help, behavioural activation, screening and watchful waiting as well as parent-led CBT. Evidence has supported the value of low intensity approaches and behavioural activation for the treatment of anxiety and depression in children and adolescents (Creswell et al., 2010; Pass, et al., 2018). These interventions also coincide with NICE Guidance for depression and anxiety (NICE Guidance, 2005; 2014) with CWPs being best placed to deliver step 2 recommended treatments. Overall, results provide promising evidence for the effectiveness of low-intensity CBT treatment for mild to moderate anxiety and depression in children and adolescents. Findings can also be interpreted with a degree of confidence given the adequate power that was obtained prior to commencement of the evaluation.

## Limitations and future investigation

One obvious limitation is that this evaluation did not employ a control sample making it difficult to make concrete conclusions regarding treatment effectiveness. That said, this evaluation does provide promising evidence for the effectiveness of low-intensity CBT delivered by CWPs within a real-world setting.

It is noted that the current sample of five trainee CWPs in a North East of England CAMHS service may not represent the outcomes of other services. Furthermore, the impact of geographical location on clinical outcome was not explored as part of this evaluation but may have influenced findings e.g., types of referrals made to CWPs. Any future evaluation should attempt to gather qualitative data from service users to ascertain the overall experiences of service users accessing CWPs in CAMHS.

A large percentage of GBO data was missing due to recording issues at the start of the evaluation period. Hence only 28 pre and post GBO completed in the later part of the evaluation are recorded. This means that extreme caution should be exercised when interpreting these findings in relation to GBOs. This has been acknowledged as a limitation that has been rectified for the next round of evaluation.

It is further acknowledged that this study did not look specifically at the diverse nature (ethnicity, language, disability status and gender) and social economic status of the participants. It is felt this would enrich future studies.

## Conclusion and recommendations

1. Expansion of the CWP service should be considered and led by the NHS senior management team to improve access to these evidence-based interventions.
2. Referral criteria for CWP intervention should be effectively communicated to the wider CAMHS team to ensure appropriate referrals as part of a stepped care model.
3. CWPs to continue to embed outcome measures into their clinical practice and support MDT staff within CAMHS to do so as part of the CYP-IAPT agenda.
4. Re-evaluation should be conducted on an annual basis to ensure ongoing effectiveness.
5. Future evaluation should be aimed at establishing the effectiveness of qualified CWPs and looking at particular interventions such as Parent-Led CBT.

These recommendations have been included in a service evaluation action plan submitted to the NHS Trust Clinical Audit and Effectiveness Team responsible for implementation of the action plan.

## Key bullet points

1. In recent years referrals to Child and Adolescent Mental Health Services have surged. In order to improve access to evidence based psychological therapies for children and young people, the government has focused on developing new professions, such as the Children’s Wellbeing Practitioner (CWP), to deliver low intensity approaches.
2. Evaluative data for the effectiveness of low intensity cognitive behavioural therapy interventions delivered by CWPs is sparse.
3. Findings from this evaluation support the potential value of low intensity cognitive behavioural therapy interventions delivered by CWPs in reducing anxiety and depression.
4. Recommendations will include the development and expansion of the CWP role and CWP Services and the need for further evaluative data.
